# Prognostic value of a three-dimensional dynamic quantitative analysis system to measure facial motion in acute facial paralysis patients

**DOI:** 10.1186/s13005-020-00230-6

**Published:** 2020-07-18

**Authors:** Yang Zhao, Guodong Feng, Haiyan Wu, Surita Aodeng, Xu Tian, Gerd Fabian Volk, Orlando Guntinas-Lichius, Zhiqiang Gao

**Affiliations:** 1 Department of Otolaryngology, Peking Union Medical College Hospital, Chinese Academy of Medical Sciences and Peking Union Medical College, No. 1 Shuaifuyuan Wangfujing, Dongcheng District, Beijing, 100730 China; 2 ENT-Department and Facial Nerve Center Jena, Jena University Hospital, Jena, Germany

**Keywords:** Bell’s palsy, Facial palsy, Three-dimensional, Prognosis, Objective

## Abstract

**Background:**

To investigate the prognostic value of a three-dimensional dynamic quantitative analysis system to measure facial motion (3D ASFM) in acute facial palsy patients and compare it with subjective grading methods and electroneurography.

**Methods:**

We continuously recruited 37 patients with acute (< 1 month) Bell’s palsy. An integrated evaluation of facial palsy was performed for each patient. The integrated evaluation included the House-Brackmann grading system (H-BGS), Sunnybrook Facial Grading System (SFGS), electroneurography and three-dimensional objective measurements. Then, the entire set of evaluations were repeated for each patient 1 month later. The patients were followed up monthly until recovery or for up to more than 6 months. We adopted the SFGS and H-BGS as the representative subjective grading system and final criteria for recovery. Poor recovery was defined as an SFGS score less than 70 or H-BGS score higher than II.

**Results:**

Multiple regression analysis was performed to find the best prognostic indicators. In less than 1 month from onset, ENoG had the highest prognostic value. However, in the second month from onset, the results of SFGS and 3D ASFM were identified as the best prognostic parameters, and a prediction formula with a determination coefficient of 0.673 was established. The receiver operating characteristic curves revealed that a gross score of the 3D ASFM less than 31 in the first evaluation and 49 in the second evaluation had higher sensitivity and specificity to predict poor recovery.

**Conclusions:**

In different phases of Bell’s palsy, the best predictor of prognosis is different. ENOG is the most effective predictor of the prognosis in the first month after onset. In the second month after onset, the combination of SFGS and 3D ADSM is considered to be the best prognostic predictor.

## Background

Facial palsy, which has various causes, is the most common cranial nerve lesion in clinical practice. The treatment strategy and prognosis can be affected by the evaluation of facial nerve function, which is often performed using facial nerve grading systems. At present, facial nerve grading systems can be roughly divided into subjective grading systems and objective grading systems [1]. The globally used House-Brackmann grading system (H-BGS) is a typical example of a subjective grading system [2]. In addition, some more precise systems, such as the Sunnybrook Facial Grading System (SFGS) [3] and Facial Nerve Grading System 2.0 [4], have been introduced by other authors. The advantages of a subjective grading system are obvious; they are intuitively simple, convenient, and inexpensive. However, because a subjective grading system depends largely on the observer’s experience, interobserver variability is high. Further, subtle changes in facial motion cannot be distinguished [5]. In regard to clinical research, subjective grading systems are far from meeting the requirements. As Neely said, “Clinical practice is generally qualitative and flexible, whereas clinical research is distinctly quantitative and rigidly fixed to a written protocol” [3].

Objective grading systems can overcome the shortcomings of subjective ones. Several objective grading instruments using different mechanisms have been designed to measure facial motions or just one facial expression [6–8]. However, none of them has been widely accepted and applied. Based on our experience with previous objective grading systems, we designed a three-dimensional dynamic quantitative analysis system for facial motion (3D ASFM) [9]. Its aim is to perform static and dynamic analyses within several seconds and to provide a comprehensive set of parameters, including movement direction, distance, velocity, acceleration, maximal velocity, and maximal acceleration. In previous experiments in healthy volunteers and patients, it was shown to be accurate and reliable. However, its predictive value for facial paralysis is unknown. The aim of this study was to compare the prognostic value of a three-dimensional dynamic quantitative analysis system for facial motion in facial paralysis patients with different facial nerve grading methods.

## Methods

### Patients

Patients seeking treatment at the ENT Department of a tertiary referral center because of sudden-onset unilateral facial palsy between April 2015 and April 2019 were examined. Patients with traumatic causes, infection of the ear, recurrent attacks of facial paralysis and inability to perform facial expressions as instructed were excluded. Patients with coexisting diseases, such as diabetes mellitus and cerebrovascular diseases, were also excluded. The factors of age, gender, and side of paralysis that were not associated with facial grading were not considered in the exclusion criteria. The diagnosis was confirmed by clinical examination. Radiological and audiological tests were performed if patients complained of hearing loss or non-recovery of facial motion 2 months after onset. The interventions provided to the patients were protective eye care, including the application of artificial tear eye drops for daytime use and eye ointment for nighttime. Full doses of steroids and antiviral agents were given to all patients. On admission, all the patients were evaluated using the 3 types of measurements in the following text. Then, the evaluation was repeated in all patients one month later. The patients were followed up monthly until recovery or for up to 6 months.

### 3D ASFM

#### Measuring instruments

**Fig. 1:**
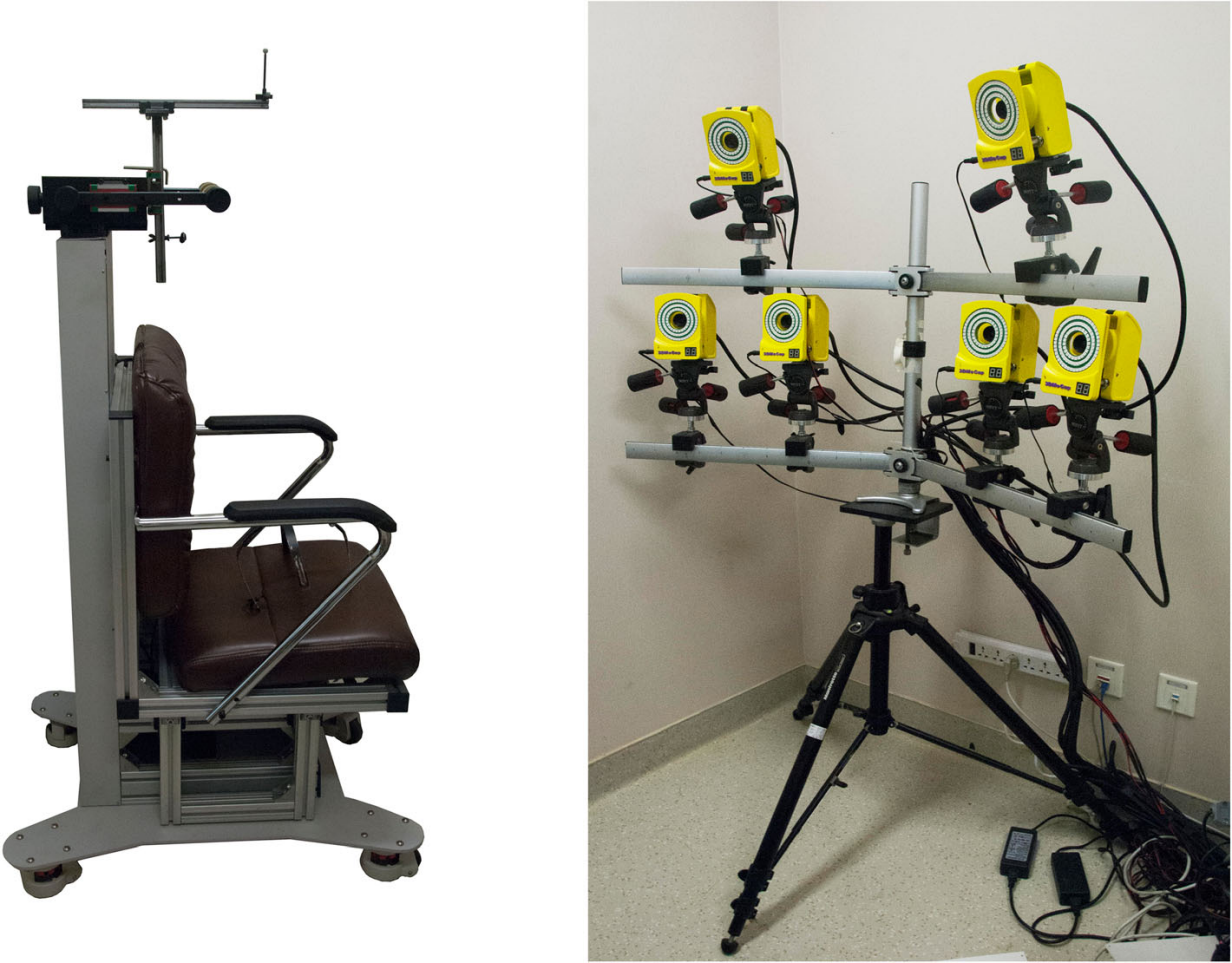
Overview of the three-dimensional dynamic quantitative analysis system to measure facial motion

The 3D ASFM is composed of the following three parts: image capture, data analysis, and output (see Fig. 1). The 3D reconstruction is based on multiview stereo vision, which has the advantage of low matching error and high accuracy. Six cameras in the image capturing portion are placed in the shape of a symmetrical “L” on a tripod to ensure that every reflective point on the patient is detected by at least three cameras. The object’s signal captured by the cameras during measurement is matched to the reference data established during calibration. The spatial location, relationship, and displacement in every frame of each landmark can then be calculated [9].

**Fig. 2:**
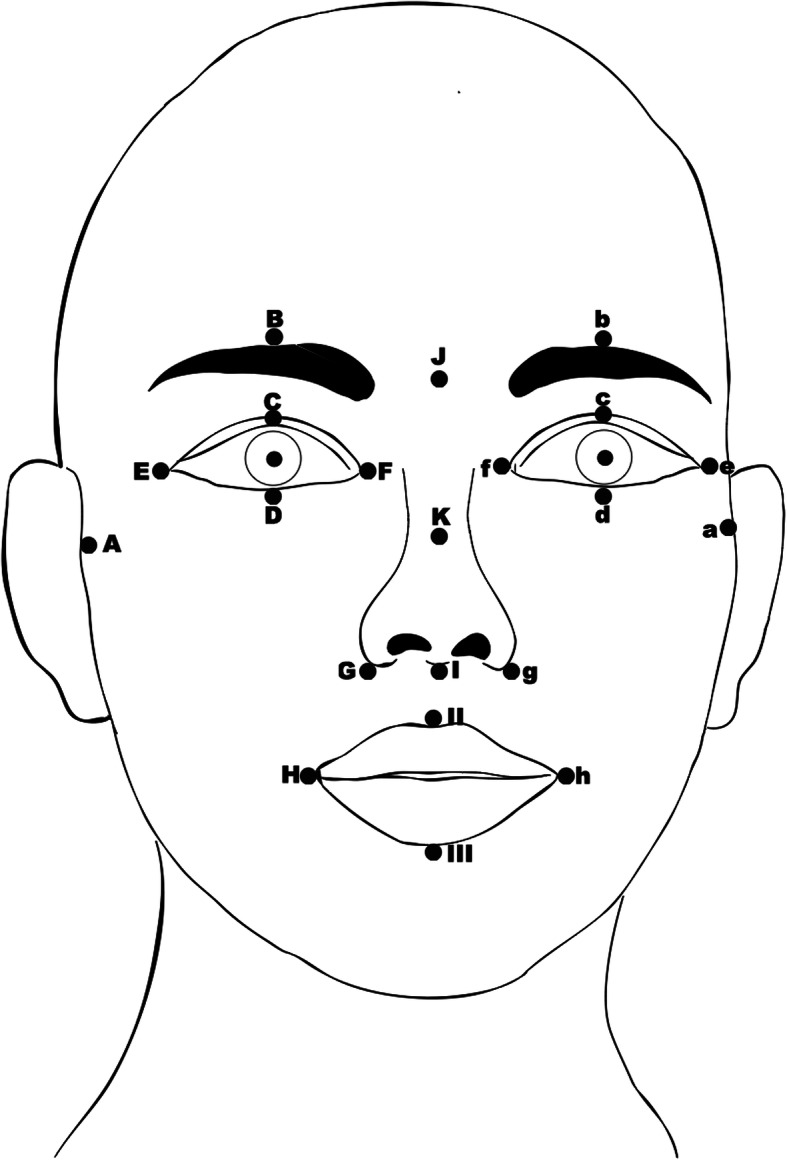
Observational points on the face. A/a: tragus parallel to the upper wall of the external acoustic canal, B/b: central position above the eyebrow, C/c: center of the upper eyelid, D/d: center of the lower eyelid, E/e: angulus oculi temporalis, F/f: angulus oculi medialis, G/g: ala of the nose, H/h: corner of the mouth, I: root of the columella nasi, J: center of eyebrows, K: bony–cartilaginous junction along the nasal dorsum, II: philtrum, and III: center of the lower lip

To fully evaluate every branch of the facial nerves, we originally designed 21 observational points on the face as follows: A/a, the tragus parallel to the upper wall of the external acoustic canal; B/b, central position above the eyebrow; C/c, center of the upper eyelid; D/d, center of the lower eyelid; E/e, angulus oculi temporalis; F/f, angulus oculi medialis; G/g, ala of the nose; H/h, corner of the mouth; I, root of the columella nasi; J, center of the eyebrows; K, bony-cartilaginous junction along the nasal dorsum; II, philtrum; and III, center of the lower lip (Fig. 2). Every observational point is labeled with a disposable reflective marker, the center of which is the measurement focus.

#### Facial expressions

The facial expressions we evaluated included brow elevation, gentle eye closure, open mouth smile, snarl, and lip pucker. Although there is no gold standard for grading regional movements, we adopted the SFGS as the representative subjective grading system and final criteria. The SFGS rates the voluntary movement symmetry of the following five standard expressions: brow lift, gentle eye closure, open mouth smile, snarl, and lip pucker.

#### Measurement procedure

**Fig. 3:**
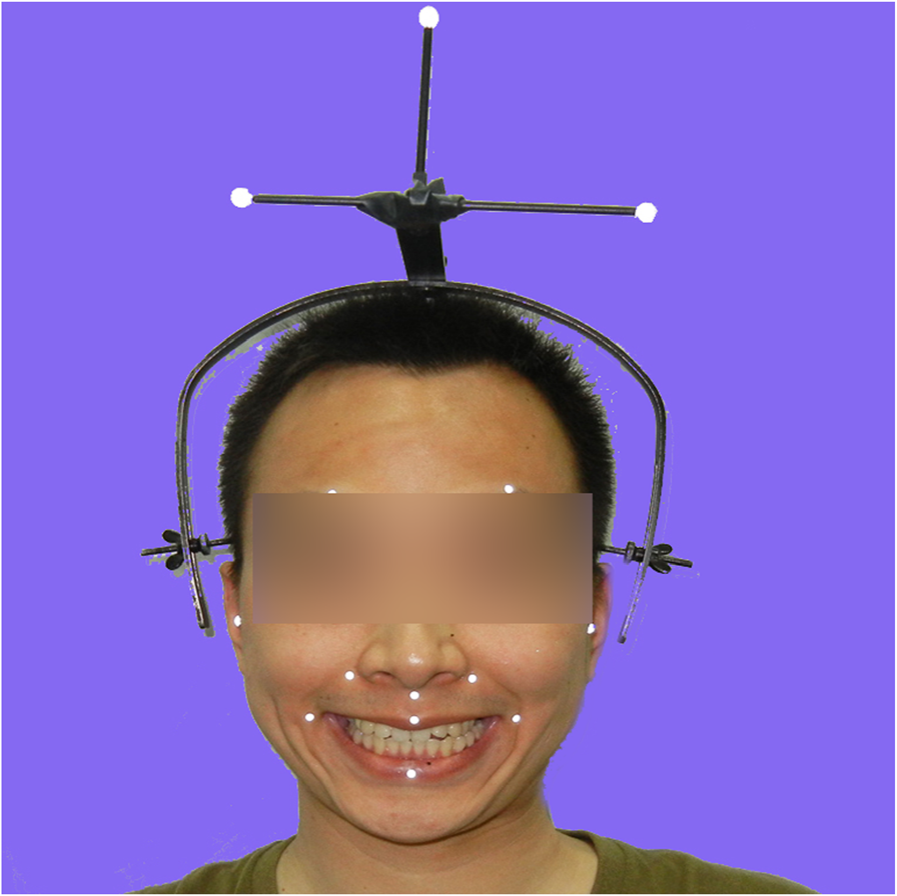
A reference helmet fixed to the head by attachment to the skin above the mastoid region, while the posterior end of the helmet was attached to the occipital protuberance

Before the test, every patient was asked to clean his/her face and remove any reflective objects from it. Adhesive reflective points were placed in precise positions on the resting face. A reference helmet (Fig. 3) described in a previous article [9] was used as a reference coordinate system that enabled the patient to freely move his/her head during the test and was firmly fixed to the head. The lateral end of the helmet was rigidly attached to the skin above the mastoid region, while the posterior end of the helmet was firmly attached to the occipital protuberance. The patients were then verbally instructed to perform five standard facial movements. While the 3D ASFM captured the markers’ trajectory, a high definition camera simultaneously recorded all the facial expressions. It took less than 1 min to perform the entire measurement. The overall time from placing the reflective points on the face to outputting the result was less than 5 min for each patient.

#### Symmetry ratio and scores

The symmetry analysis includes regional and gross analyses. Symmetry ratios (SRs) can be calculated by comparing the movement of bilateral observational points. The symmetry ratio in this study is defined as the ratio of motion on the paralyzed side to that on the contralateral side. When the brow is analyzed, the maximal moving distance (MMD), maximal moving speed (MMS), and maximal moving acceleration (MMA) of point B/b on the paralyzed side are compared to those on the intact side. Then, the symmetry ratio of maximal moving distance on the paralyzed side is divided by that on the contralateral side. The process is the same for the other facial areas; point C/c, which represents the eye area, is analyzed using eye closure, point G/g in the nasolabial fold is analyzed using the snarl, and point H/h is analyzed using the open mouth smile and lip pucker. When analyzing the face at rest, the distance or angle between the observation points is measured. There are 5 pairs of resting parameters compared between sides: G-H/g-h, G-I/g-I, C-D/c-d, E-H/e-h, and ∠CED/∠ced. Since the scale of resting parameters on the paralyzed side can be either larger or smaller than that on the contralateral side, the symmetry ratio of the 5 pairs of resting parameters was calculated by dividing the results on the right side by those on the left side. The ratio of healthy subjects obtained prior to this study was used to establish a normal reference value. An abnormal pair of resting parameters received a score of 4, and a score of 20 was assigned if all resting parameters were abnormal.

Having obtained the symmetry ratios in different areas with different facial expressions, the gross score of the 3D ASFM can be calculated using the equation: score = 0.7 × D + 0.3 × V-A. In the formula, D is the average symmetry ratio associated with the maximal moving distance of corresponding points expressed as a percentile, V is the average symmetry ratio of the maximal moving velocity of corresponding points, and A is the total resting score. We generated the formula with reference to the Sunnybrook grading system. Different constants were weighted according to their importance and the regression analysis results based on other grading systems in the preliminary experiment.

### Subjective grading

Before the subjective evaluation, a clinician was given written instructions for each subjective grading system. He assessed each patient at the same time as the 3D ASFM test using the H-BGS and SFGS. H-BGS scores range from normal (I) to total paralysis (VI), whereas a function score ≥ II is considered to indicate good recovery. SFGS is a regionally weighted grading system, which includes an assessment of resting symmetry, symmetry of voluntary movement and synkinesis.

### Electrophysiological measurement

We adopted electroneurography (ENoG) to perform electrophysiological measurements. The ground electrode was placed on the patients using the wrist electrode.

All ENoG was performed by the same doctor, and the same technique was used for all patients. A signal processor (Schwarzer Topas, Natus Europe GmbH, München, Germany) was used to amplify and record the response from disc-type recording surface electrodes, with filter settings of 20 to 3000 Hz and a sensitivity of 1 mV. The recording active electrode was placed in the suborbital, nasolabial fold and mouth corner regions on the stimulated side, the average of which was used as the final result. The reference electrode was placed on the nasolabial fold on the opposite side of the face. The stimulating electrode pair was placed, with the negative pole pointing forward, around the main trunk of the facial nerve on the skin over the stylomastoid foramen just behind the earlobe. To perform BR, the recording active electrodes were placed under the orbital rim, whereas the reference electrodes were placed laterally proximal to the active one.

### Statistical analysis

We used a multivariate regression method to establish the relation between the results of the three measurements and the final SFGS score after six months. The statistics were calculated with IBM SPSS Statistics (version 22). The Green formula *n* ≥ 8 (1- R^2^)/R^2^ + (m-1) was used to decide whether the number of patients satisfied the requirement of the test [10]. In the Green formula, the minimum number of subjects (n) is determined by the number of predictors (m) and determination coefficients (R^2^). To further determine the prognostic value of 3D ASFM, receiver operating characteristic (ROC) curves were constructed to discriminate between patients with good recovery and bad recovery in the final evaluation; the area under the ROC curve (AUC) was calculated with the 95% confidence interval (95% CI). The AUC demonstrates the overall discriminative power. The accuracy of the AUC is classified as low if the area is 0.5–0.7, moderate if the area is 0.7–0.9, and high if the area is > 0.9. ROC curves were generated with OriginPro 9.0. *P* values were considered significant if they were less than 0.05.

## Results

### Basic information

Fifty-six patients with acute unilateral facial paralysis were initially considered eligible for the study. Since the prognosis of Bell’s palsy and Ramsay-Hunt syndrome differs, 12 patients diagnosed with Ramsay-Hunt syndrome were excluded from this study. One patient was later diagnosed with middle ear cholesteatoma, 4 failed to return to the hospital and 2 decided to drop out. The rate of loss to follow-up was 7/44 (16%). There were no significant differences in the initial evaluations of those who completed the study compared to the evaluations of those who were lost to follow-up (*P* > 0.05).

Of the 37 remaining patients with Bell’s palsy, 26 were male and 11 were female. The average age was 40 ± 15 years. All were diagnosed with Bell’s palsy. The right and left sides were involved in 18 and 19 patients, respectively. There was no significant difference concerning the degree of facial palsy with regard to side or gender in either the initial visit (*P* > 0.05) or the final visit (*P* > 0.05). Additionally, age was not correlated with the degree of facial paralysis in each evaluation (*P* > 0.05). Table 1 shows the results of the univariate regression analysis of basic variables, none of which were significantly correlated with poor recovery.

**Table 1:** Univariate analysis of prognostic factors for poor recovery (predictors for poor recovery were defined as HBGS II or better after 6 months)

	Odds ratio	95% confidence interval	*P* value
Gender (male)	1.658	0.285–9.639	0.574
Side (left)	0.693	0.153–3.139	0.635
Age, yr			
< 30	0	0	0.999
30–60	1(ref)	0.051–5.553	0.597
> 60	0.587	0.051–5.553	0.597
Pain (yes)	4.583	0.930–22.585	0.061
Presbycusis (yes)	1.048	0.171–6.416	0.960

**Fig. 4:**
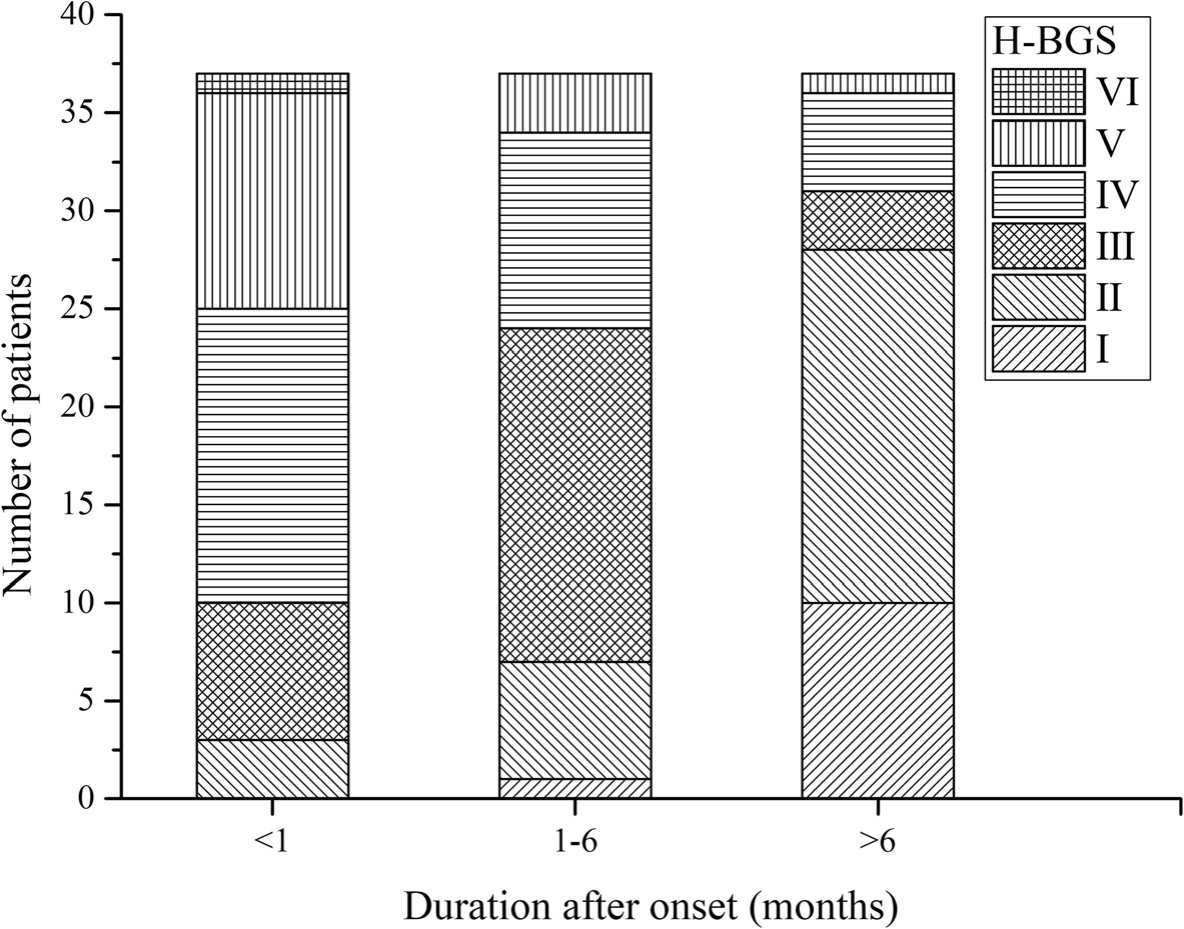
The results of the first, second and third H-BGS

**Fig. 5:**
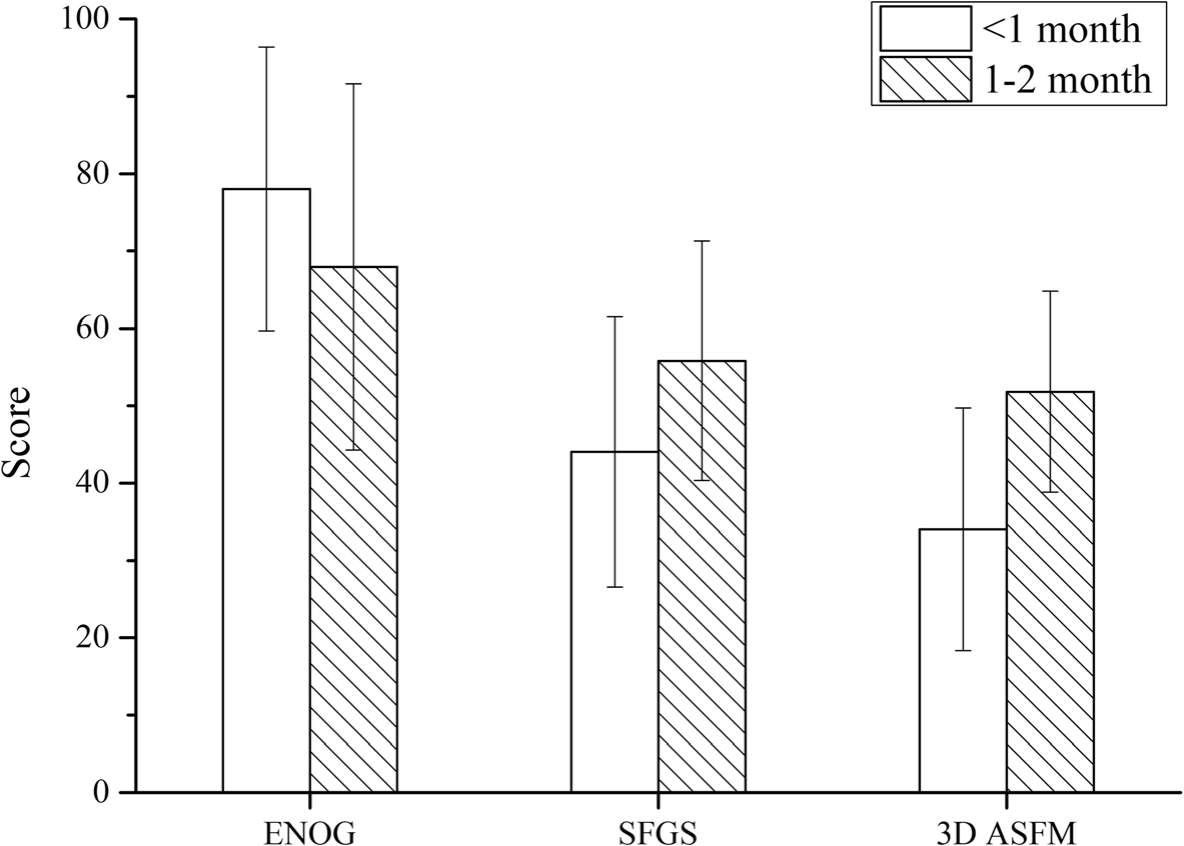
The results of the first, second and third ENOG, SFGS and 3D ASFM

Figure 4 demonstrates the results of the first, second and third H-BGS. The corresponding results of ENOG, the SFGS, and the 3D ASFM are shown in Fig. 5. We can conclude that there were significant differences between the first and second test results. The Wilcoxon rank test was employed to test for differences between the first and second H-BGS (*P* = 0.000). There were significant differences between the results of ENOG, the SFGS, and the 3D ASFM using the paired *t* test (P = 0.000). Furthermore, a significant difference existed between the second and final visits with respect to the H-BGS and SFGS (P = 0.000).

### Comparison of prognostic value

In the first month after onset, the determination coefficients (R^2^) with the final SFGS scores for different grading systems were as follows: ENOG *R*^*2*^ *=* 0.482 (*P* = 0.000), SFGS *R*^*2*^ *=* 0.395 (*P* = 0.000) and 3D ASFM *R*^*2*^ *=* 0.330 (*P* = 0.000). The stepwise regression analysis established a regression relation based on the ENOG and the final SFGS scores. The following predictive formulas were created: *y* =132.112–0.699 *x*, in which *y* was the final SFGS score and *x* was the facial nerve degenerative rate estimated by ENoG (expressed in 100× %). The determination coefficient of the formula was 0.482, which means that 48.2% of the final SFGS scores could be explained by the result in less than one month using this formula.

In the second month after onset, the determination coefficients (R^2^) for different grading systems were as follows: ENOG *R*^*2*^ *=* 0.478 (*P* = 0.000), SFGS *R*^*2*^ *=* 0.468 (*P* = 0.000) and *R*^*2*^ *=* 0.609 (*P* = 0.000). Using the step-by-step regression method, the results of SFGS and 3D ASFM were identified as the best prognostic parameters. Then, the following predictive formula was created: *y* =13.457 + 0.384 *x*_1_ + 0.823 *x*_2_, in which y was the score on the SFGS after six months, *x*_1_ was the SFGS score in the second month, and *x*_2_ indicated the simultaneous results of the 3D ASFM. The determination coefficient of the formula was 0.673, which means that 67% of the final SFGS scores could be explained by the SFGS and 3D ASFM after 1 month.

**Fig. 6:**
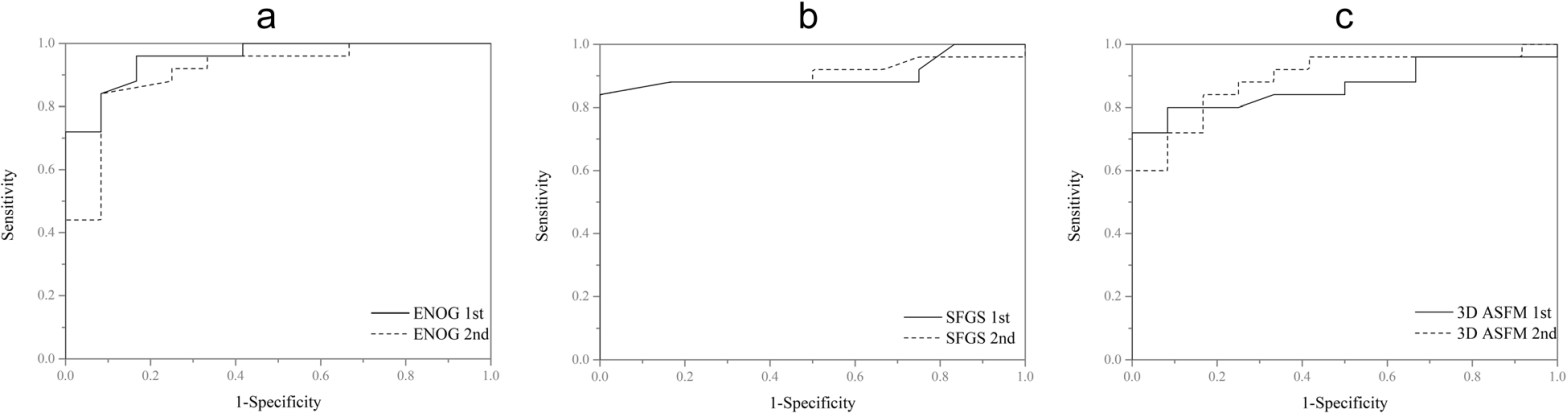
The receiver operating characteristic curves for the first and second evaluation using the Sunnybrook Facial Grading System. 1st:first evaluation, continuous line; 2nd: second evaluation, dashed line. **a** Electroneurography. **b** Sunnybrook Facial Grading System. **c** Three-dimensional dynamic quantitative analysis system for facial motion (3D ASFM)

**Fig. 7:**
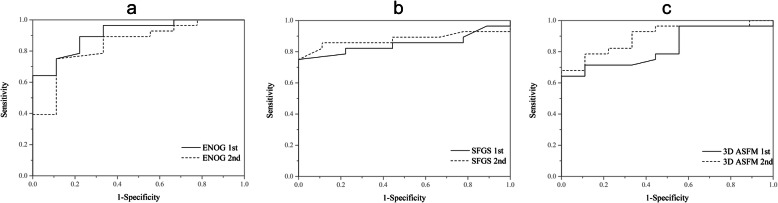
The receiver operating characteristic curves for the first and second evaluations using the House-Brackmann Grading System. 1st: first evaluation, continuous line; 2nd: second evaluation, dashed line. **a** Electroneurography. **b** Sunnybrook Facial Grading System. **c** Three-dimensional dynamic quantitative analysis system for facial motion (3D ASFM)

We classified the final SFGS scores ≥70 as good recovery and those < 70 as poor recovery. According to these criteria, 25 (67.6%) of the 37 patients showed a good recovery and 32.4% showed a poor recovery with SFGS < 70. The ROC curves are shown in Fig. 6, and the sensitivity and specificity of each cutoff value are listed in Table 2. In the first evaluation, the cut-off value for the 3D ASFM was 31, with an area under the curve of 0. 868(*P* = 0.006; 95% CI = 0.75-0.98). In the second evaluation, the cut-off value increased to 49, with an area under the curve of 0.893 (*P* = 0.000; 95% CI = 0.79-0.99). When a final H-BGS score ≥ II was considered as good recovery, 28 (75.7%) of the 37 patients showed good recovery. The corresponding ROC curves and the sensitivity and specificity of each cutoff value are shown in Fig. 7 and Table 3.

**Table 2:** Shows the sensitivity and specificity of each cut-off value using the Sunnybrook Facial Grading System

	Cut-off	Specificity (%)	Sensitivity (%)	AUC
ENOG (1st)	87	92	84	0.955
ENOG2 (2nd)	80	92	84	0.910
SFGS (1st)	40	100	84	0.903
SFGS2 (2nd)	51	100	84	0.908
3D ASFM (1st)	31	92	80	0.868
3D ASFM (2nd)	49	83	84	0.893

AUC: area under the ROC, 1st: first evaluation; 2nd: second evaluation; ENOG: electroneurography; SFGS: Sunnybrook Facial Grading System; 3D ASFM: three-dimensional dynamic quantitative analysis system for facial motion

**Table 3:** Shows the sensitivity and specificity of each cut-off value using the House-Brackmann Grading System

	Cut-off	Specificity (%)	Sensitivity (%)	AUC
ENOG (1st)	91	78	90	0.910
ENOG2 (2nd)	80	89	75	0.845
SFGS (1st)	36	78	82	0.849
SFGS2 (2nd)	48.5	89	86	0.879
3D ASFM (1st)	31	89	71	0.827
3D ASFM (2nd)	49	89	79	0.897

AUC: area under the ROC, 1st: first evaluation; 2nd: second evaluation; ENOG: electroneurography; SFGS: Sunnybrook Facial Grading System; 3D ASFM: three-dimensional dynamic quantitative analysis system for facial motion

## Discussion

This study was designed to determine the prognostic value of a three-dimensional dynamic quantitative analysis system to measure facial motion (3D ASFM) and to establish a reliable model to predict the long-term outcome of acute facial palsy. This study is unique in that it is the first study, to the best of our knowledge, to investigate the prognostic value of a three-dimensional objective grading system for facial nerve paralysis. We innovatively integrated the result of the three-dimensional objective test in the prediction formula, which increases its predictive value for a certain recovery period.

It is believed electrophysiological examinations can reflect the prognosis of facial paralysis in the acute phase. On the 20th day after the onset of facial paralysis, the sensitivity and specificity of ENOG are both above 86%. A statistically significant correlation was observed between ENOG with a CMAP difference ≥ 75% on the 20th day and a poor prognosis [11]. However, when different grading methods and recovery criteria are adopted, the threshold values for predicting facial palsy recovery are also different [11–13]. In general, most studies have recommended a bilateral difference from 70 to 90% as the cut-off value. Some researchers tried to establish a formula containing ENOG results to estimate the prognosis of facial paralysis, but the coefficient of determination was only 0.45 [14]. In our study, the ENOG produced the best prognostic value in patients with acute facial paralysis within one month after clinical onset. The coefficient of determination was 0.482, meaning that 48.2% of the SFGS score after half a year can be explained by this formula. We classified patients with a final SFGS score above 70 or HBGS ≥II as having a good recovery. Facial degeneration greater than 87 or 91, respectively, in the first month after onset was considered to indicate a poor prognosis. There is no doubt that recovery rates in a facial palsy study are affected by the choice of grading systems [15].

After more than one month of facial palsy, the ENOG results gradually diverge from the clinical symptoms due to neuromuscular junction and muscle function degeneration. Recovery according to ENOG may lag behind clinical recovery for several months [16], which might explain the decrease in sensitivity in the second month. Electromyography has a greater correlation with prognosis in this period, but it is not widely performed because of its invasive quality, qualification and interoperator variance. On the other hand, subjective evaluation systems are more valuable during this period. Marsk et al. [17] used the SFGS to predict the prognosis of facial palsy 3 days, 2 weeks, and 1 month after onset of facial palsy. At days 11 to 17 and at 1 month, the SFGS was the only significant predictive variable, which showed the most accurate prediction of nonrecovery at 1 month. It should be noted that when using the subjective evaluation system for prognostic evaluation, it is the worst score rather than the initial score that significantly correlates with the final outcome [18]. Fujiwara [19] recommended that four weeks after the onset is the best time for evaluation, followed by the second week. In this study, the second evaluation of all patients did not occur on the same day after onset. Because patients presented to our hospital on different days after onset, some arrived near the end of the first month. Instead, the patients’ second tests were performed 2 to 4 weeks after their initial visit, which was close to 6 weeks after onset. This might account for the difference in predictive values between this study and other reports.

The correlation between 3D measurement techniques based on motion capture technology and previous facial grading systems (e.g., SFGS, H-B, Yanagihara) has been validated [20–22]. However, the prognostic value of the three-dimensional objective measurement is currently unknown, and no relevant reports have been published. The results of this study demonstrated that subjective evaluation systems can be used as independent prognostic predictors following ENOG in the first month. The coefficient of determination could increase when the ENoG results are taken into account. When the SFGS score after 6 months was used as the final variable, 3D ASFM was not a good prognostic predictor in the first month. In the second month, the combination of 3D ASFM and SFGS generated a useful formula to predict the outcome of the SFGS score several months later. The reason for this might lie in the instability of muscular motion in the first month. On the other hand, it might be due to measurement error. A precision test revealed that the maximal dynamic measuring error of 3D ASFM was 0.005800 m/s, which is an amount that can be ignored in normal facial motion. However, when facial palsy is severe, this error might confuse the test result.

We followed several patients using 3D ASFM and found it was sensitive to facial nerve recovery sooner than clinical observation. However, the weight of the moving velocity variable was lower than that of the moving distance variable because (1) the capture frequency of the 3D systems was 30 Hz, which might influence the velocity calculation and (2) velocity might fluctuate sharply within the motion process. In a previous study, we also found that the velocity documented in the lower face was less stable than that in the upper face. Neely found that the lip puckering facial action region is the location of the majority of variances [3].

As stated above, the disadvantages of subjective grading systems include poor accuracy and reliability [23]. Hence, the aim of an objective grading system is not to perfectly match and replace subjective grading systems. Poor correlation with them does not indicate that the objective system is not precise. In contrast, in the future, we might need to adjust the metrics of the subjective grading systems to correlate better with the objective results. An objective grading system can overcome the poor interobserver reliability of a subjective grading system. It is much more precise and hence should be the ultimate gold standard for facial nerve grading [24]. However, no matter how simple an objective grading system is to use, it cannot become a substitute for subjective grading systems, because the main goal of facial nerve recovery is to allow expression recognition by other people, which is the essence of what a subjective grading system measures. An ideal treatment plan would combine subjective and objective grading systems and use each at the optimal time.

The disadvantages of 3D-ASFM are that it currently requires a specific instrument. It is not as convenient as traditional subjective grading systems and some simple objective scales. However, ENoG requires specific instruments, training and time. Additionally, in contrast to 3D-ASFM and subjective grading systems, it is painful for the patient. We will endeavor to make it smaller and more automatic. Perhaps in the future, an objective grading system could be as small and intelligent as a cellphone.

## Conclusion

In patients with acute facial palsy, electrophysiological examination was the best predictor of recovery of the facial palsy. After one month, the combination of SFGS and the three-dimensional dynamic subjective evaluation was the best prognostic predictor.

## Supplementary information

**Additional file 1.**

## Data Availability

The datasets supporting the conclusions of this article are included within the article and its additional file.

## Abbreviations

3D ASFM: Three-dimensional dynamic quantitative analysis system to measure facial motion
ENOG: Electroneurography
SFGS: Sunnybrook Facial Grading System
H-BGS: House-Brackmann grading system

## References

[1] Chee GH, Nedzelski JM (2000). Facial nerve grading systems. Facial Plast Surg..

[2] House JW, Brackmann DE (1985). Facial nerve grading system. Otolaryngol Head Neck Surg..

[3] Neely JG, Cherian NG, Dickerson CB, Nedzelski JM (2010). Sunnybrook facial grading system: reliability and criteria for grading. Laryngoscope..

[4] Vrabec JT, Backous DD, Djalilian HR, Gidley PW, Leonetti JP, Marzo SJ (2009). Facial nerve grading system 2.0. Otolaryngol Head Neck Surg..

[5] Kang TS, Vrabec JT, Giddings N, Terris DJ (2002). Facial nerve grading systems (1985–2002): beyond the House-Brackmann scale. Otol Neurotol..

[6] Frey M, Jenny A, Giovanoli P, Stussi E (1994). Development of a new documentation system for facial movements as a basis for the international registry for neuromuscular reconstruction in the face. Plast Reconstr Surg..

[7] Wachtman GS, Cohn JF, VanSwearingen JM, Manders EK (2001). Automated tracking of facial features in patients with facial neuromuscular dysfunction. Plast Reconstr Surg..

[8] Sawyer AR, See M, Nduka C (2010). Quantitative analysis of normal smile with 3D stereophotogrammetry--an aid to facial reanimation. J Plast Reconstruct Aesthetic Surg..

[9] Feng G, Zhao Y, Tian X, Gao Z (2014). A new 3-dimensional dynamic quantitative analysis system of facial motion: an establishment and reliability test. PLoS One..

[10] Green SB (1991). How many subjects does it take to do a regression analysis. Multivar Behav Res..

[11] Ozgur A, Semai B, Hidir UU, Mehmet Fatih O, Tayfun K, Zeki O (2010). Which electrophysiological measure is appropriate in predicting prognosis of facial paralysis?. Clin Neurol Neurosurg..

[12] Khedr EM, Abo El-Fetoh N, El-Hammady DH, Ghandour AM, Osama K, Zaki AF (2018). Prognostic role of neurophysiological testing 3-7 days after onset of acute unilateral Bell's palsy. Neurophysiol Clin..

[13] Chow LC, Tam RC, Li MF (2002). Use of electroneurography as a prognostic indicator of Bell's palsy in Chinese patients. Otol Neurotol..

[14] Ushio M, Kondo K, Takeuchi N, Tojima H, Yamaguchi T, Kaga K (2008). Prediction of the prognosis of Bell's palsy using multivariate analyses. Otol Neurotol..

[15] Berg T, Marsk E, Engstrom M, Hultcrantz M, Hadziosmanovic N, Jonsson L (2009). The effect of study design and analysis methods on recovery rates in Bell’s palsy. Laryngoscope..

[16] Slattery W (2014). The Facial Nerve.

[17] Marsk E, Bylund N, Jonsson L, Hammarstedt L, Engstrom M, Hadziosmanovic N (2012). Prediction of nonrecovery in Bell’s palsy using Sunnybrook grading. Laryngoscope..

[18] Takemoto N, Horii A, Sakata Y, Inohara H (2011). Prognostic factors of peripheral facial palsy: multivariate analysis followed by receiver operating characteristic and Kaplan-Meier analyses. Otol Neurotol..

[19] Fujiwara T, Hato N, Gyo K, Yanagihara N (2014). Prognostic factors of Bell's palsy: prospective patient collected observational study. Eur Arch Otorhinolaryngol..

[20] Katsumi S, Esaki S, Hattori K, Yamano K, Umezaki T, Murakami S (2015). Quantitative analysis of facial palsy using a three-dimensional facial motion measurement system. Auris Nasus Larynx..

[21] Sawai N, Hato N, Hakuba N, Takahashi H, Okada M, Gyo K (2012). Objective assessment of the severity of unilateral facial palsy using OKAO vision(R) facial image analysis software. Acta Otolaryngol..

[22] Haase D, Minnigerode L, Volk GF, Denzler J, Guntinas-Lichius O (2015). Automated and objective action coding of facial expressions in patients with acute facial palsy. Eur Arch Otorhinolaryngol..

[23] Lee LN, Susarla SM, Hohman MH, Henstrom DK, Cheney ML, Hadlock TA (2013). A comparison of facial nerve grading systems. Ann Plast Surg..

[24] Fattah A, Gurusinghe A, Gavilan J, Hadlock T, Marcus J, Marres H (2015). Facial Nerve Grading Instruments: Systematic Review of the Literature and Suggestion for Uniformity. Plast Reconstruct Surg..

