# Supplementary material for: Prognostic value of a three-dimensional dynamic quantitative analysis system to measure facial motion in acute facial paralysis patients
Source: Head Face Med. 2020 Jul 18;16:15. doi: 10.1186/s13005-020-00230-6 (PMC7368680; doi:10.1186/s13005-020-00230-6)
Supplement: Supplementary file 1 — Additional file 1. [file 13005_2020_230_MOESM1_ESM.docx]

| Sequence | pain | hyperacusis | Sex（1=M | age | ENOG 1st | HBGS 1st | SFGS 1st | 3DASFM 1st | ENOG 2nd | HBGS 2nd | SFGS 2nd | 3DASFM 2nd | HB Final | SFGS Final |
| --- | --- | --- | --- | --- | --- | --- | --- | --- | --- | --- | --- | --- | --- | --- |
| 6 | 1 | 0 | 1 | 39 | 95.9596 | 4 | 39 | 25.43083 | 95.94995 | 4 | 38 | 28.4591 | 5 | 38 |
| 18 | 0 | 0 | 1 | 49 | 100 | 5 | 26 | 7.34236 | 87.68519 | 5 | 35 | 25.58374 | 4 | 39 |
| 13 | 0 | 0 | 1 | 39 | 94.66667 | 4 | 39 | 26.2944 | 92.51785 | 3 | 47 | 44.40193 | 4 | 47 |
| 33 | 1 | 1 | 2 | 35 | 94.33333 | 5 | 34 | 13.37638 | 90.66837 | 4 | 46 | 31.25112 | 4 | 55 |
| 27 | 0 | 0 | 1 | 47 | 86.66667 | 5 | 21 | 16.12351 | 80.09399 | 4 | 44 | 34.3018 | 3 | 55 |
| 10 | 1 | 0 | 1 | 49 | 100 | 5 | 34 | 28.0914 | 100 | 4 | 46 | 47.66126 | 4 | 55 |
| 36 | 1 | 0 | 1 | 67 | 94.33333 | 5 | 29 | 33.00236 | 80.09399 | 4 | 44 | 33.19813 | 4 | 55 |
| 1 | 0 | 0 | 1 | 36 | 91.16162 | 5 | 19 | 29.68925 | 82.99383 | 3 | 51 | 48.90942 | 3 | 60 |
| 23 | 1 | 1 | 2 | 32 | 84.33333 | 5 | 27 | 13.37638 | 60.66837 | 4 | 34 | 51.25112 | 3 | 63 |
| 14 | 1 | 1 | 1 | 61 | 92.73088 | 5 | 12 | 21.77318 | 88.27778 | 5 | 33 | 52.63029 | 2 | 64 |
| 21 | 0 | 0 | 1 | 35 | 90.66667 | 4 | 31 | 16.94924 | 82.16667 | 3 | 50 | 45.90913 | 2 | 64 |
| 19 | 0 | 0 | 1 | 52 | 94.87179 | 5 | 38 | 26.18121 | 93.33333 | 4 | 51 | 38.27919 | 2 | 68 |
| 5 | 0 | 0 | 1 | 45 | 87.30159 | 5 | 21 | 16.13561 | 79.64954 | 4 | 38 | 48.70124 | 2 | 72 |
| 22 | 1 | 0 | 2 | 33 | 85.33333 | 4 | 55 | 44.6939 | 75.41667 | 3 | 58 | 68.54848 | 2 | 74 |
| 11 | 0 | 0 | 2 | 41 | 72.22222 | 3 | 43 | 55.7457 | 71.08333 | 3 | 51 | 26.47894 | 2 | 76 |
| 3 | 1 | 1 | 2 | 32 | 89.27739 | 6 | 22 | 5.83967 | 74.50088 | 5 | 26 | 49.03333 | 2 | 76 |
| 25 | 0 | 0 | 1 | 48 | 67.33333 | 3 | 51 | 48.6873 | 60 | 3 | 59 | 49.93123 | 2 | 76 |
| 20 | 0 | 0 | 1 | 31 | 93.27731 | 4 | 39 | 34.01923 | 91.4091 | 3 | 55 | 57.25112 | 2 | 80 |
| 30 | 0 | 0 | 1 | 40 | 67.33333 | 3 | 60 | 48.6873 | 60 | 3 | 55 | 59.90193 | 2 | 80 |
| 37 | 0 | 0 | 2 | 65 | 84.44444 | 4 | 47 | 23.06825 | 82.44444 | 3 | 55 | 52.17197 | 2 | 80 |
| 28 | 1 | 1 | 1 | 38 | 74.66667 | 4 | 51 | 26.2944 | 72.51785 | 3 | 60 | 51.4121 | 2 | 84 |
| 35 | 0 | 0 | 1 | 70 | 71.24183 | 4 | 50 | 41.23219 | 61.54762 | 4 | 52 | 51.70199 | 2 | 84 |
| 34 | 0 | 1 | 1 | 12 | 70 | 4 | 42 | 51.7626 | 70.63212 | 2 | 80 | 45.53019 | 2 | 84 |
| 29 | 0 | 0 | 1 | 20 | 83.27731 | 4 | 55 | 34.62139 | 81.49483 | 3 | 60 | 47.25112 | 2 | 84 |
| 2 | 0 | 1 | 2 | 33 | 85.14957 | 2 | 56 | 50.7139 | 70.32343 | 3 | 66 | 54.72149 | 2 | 84 |
| 31 | 0 | 0 | 1 | 55 | 73 | 4 | 47 | 32.14326 | 58.75 | 2 | 76 | 64.33333 | 2 | 88 |
| 7 | 0 | 0 | 1 | 9 | 31.28655 | 3 | 60 | 57.81013 | 24.7076 | 2 | 80 | 62.80193 | 1 | 96 |
| 9 | 0 | 0 | 1 | 40 | 82.51462 | 4 | 55 | 43.72057 | 59.72222 | 3 | 54 | 67.23917 | 1 | 96 |
| 15 | 0 | 0 | 2 | 44 | 33.34967 | 2 | 92 | 66.59563 | 3.174603 | 1 | 96 | 76.9537 | 2 | 96 |
| 24 | 0 | 0 | 1 | 10 | 70 | 4 | 59 | 41.7626 | 20.59649 | 2 | 68 | 69.03123 | 1 | 96 |
| 17 | 1 | 0 | 2 | 26 | 58.19571 | 2 | 76 | 40.43333 | 57 | 2 | 76 | 62.43091 | 1 | 100 |
| 8 | 0 | 0 | 1 | 48 | 71.05556 | 3 | 51 | 47.7626 | 64.11765 | 3 | 71 | 64.88657 | 1 | 100 |
| 4 | 1 | 0 | 2 | 51 | 79.52381 | 4 | 55 | 35.40388 | 18.94444 | 3 | 75 | 69.43333 | 1 | 100 |
| 26 | 0 | 0 | 1 | 73 | 74.74206 | 3 | 51 | 41.25542 | 74.18478 | 3 | 59 | 50.02723 | 1 | 100 |
| 16 | 0 | 0 | 1 | 27 | 31.60278 | 3 | 47 | 32.06839 | 31.33333 | 2 | 76 | 64.3712 | 1 | 100 |
| 32 | 0 | 1 | 2 | 34 | 45.33333 | 4 | 75 | 64.6939 | 35.41667 | 3 | 55 | 58.54848 | 1 | 100 |
| 12 | 0 | 0 | 1 | 45 | 86.66667 | 5 | 21 | 16.13561 | 80.09399 | 4 | 45 | 62.9201 | 1 | 100 |
